# Computation of fractal dimension and power draw of regrinding depending on particle size distribution

**DOI:** 10.1038/s41598-025-98950-9

**Published:** 2025-04-23

**Authors:** Iman Moradi, Ali Akbar Abdollahzadeh, Mehdi Irannajad

**Affiliations:** Department of Mining Engineering, Amirkabir University of Technology (Tehran Polytechnique), Tehran, Iran

**Keywords:** Ball mill, Power draw, Fractal, Grinding, Particle size distribution, Energy science and technology, Engineering, Materials science

## Abstract

This article uses particle size distribution to examine the fractal dimension and power draw of grinding. The tailings from dry magnetic separators, which are fed into the regrinding mill of the Gol Gohar iron ore mine, were used as the sample under study. The size of the balls, the filling particles, and the fractional ball filling have been studied as input variables, with the response variables being the fractal dimension and power draw. The results demonstrate that increasing energy usage does not increase the fractal dimension. Conversely, a direct relationship was shown between the fractal dimension and the quantity of fine particles. The self-similarity principle of fractal geometry can be used to predict the repeated process that results in variations in power draw over time. This might be the start of numerous studies that foresee the rising power draw peaks and reduce their duration by adjusting the effective parameters.

## Introduction

Early grinding and regrinding are the two stages of mineral grinding. Regrinding is an open circuit process that is only seen in mineral upgrading sections. Additionally, according to a 2019 Metso study, comminution uses 3% of global electrical energy. However, comminution accounts for 53% of the total energy used in mining. Furthermore, according to a 2023 estimate, mines would eventually use up to 12% of the world’s electrical energy, underscoring the need for comminution process optimization. Conversely, the goal of the comminution process is to get the appropriate granulation for the subsequent stages. As a result, every parameter that may achieve the required granulation while using less energy to crush the material must be taken into consideration^1–4^. The quantity of consumption and waste energy has been measured using a variety of techniques in recent years, including Bond’s law, Rettinger, Kick^3,4^, discrete element methods (DEM)^5^, fractal^6^, and other energy consumption techniques (such as Morrell^7^, Rose and Evans^8^, Moys^9^, Van Nierop et al.^10^).

To enhance the separation process and raise the metallurgical indices of grade and recovery, a concentrate/tailing (typically obtained by flotation, gravity, or magnetic separators), is regrind. Usually, 80% of the particles in regrinding are ground to a size of 25 or 30 microns^11,12^. Ball mills, Isamill, and particularly vertical agitator mills are often used when discussing regrinding. Several factors and variables have been studied concerning regrinding, including the kind of mill; the ratio of length to the diameter of the mill; sizes of input particles; dimensions of ball and pellets; solid weight percentage pulp; ball filling; speed of mill, etc. Numerous studies have looked into the factors influencing regrinding’s energy consumption. These parameters include, among others: rotation of the Bond ball mill for ores and different mill configurations^13^, vertical mills in a closed working circuit that feed from the bottom and ball mill^14^, the regular and cascading movement of balls inside the mill^15^, the Bond ball mill and agitator mill in wet and dry mode^16^, and the percentage of solid weight and the speed of continuous and discontinuous ball mills^17^. Thus, it is evident that the regrinding industry has extremely high energy consumption, which may be greatly improved and decreased by selecting the appropriate equipment and operating conditions.

In an attempt to describe the process of grinding power consumption, the early grinding mechanisms mainly considered the grinding behavior of a single particle^18^. Particle grinding phenomena, energy usage efficiency, the connection between grinding energy and particle size, and the material’s mechanical properties during grinding are the key areas of study for single particle grinding^19,20^. The aim is to develop a model of the whole particle grinding system by examining the energy consumption law and mechanical behavior of a single particle grinding, therefore expanding the conclusion to the grinding process of a large number of particles^21^. The Kick theory is based on the elastic deformation of the volume, the Bond theory is based on cracks and the propagation of cracks, and the Rittinger theory, which is based on the creation of the new surface area created by the fracture of particles, are the three fundamental theories for calculating energy consumption^22^. More precise techniques for estimating energy use were suggested in the following once the correlation coefficients were adjusted^23,24^. Variations in surface area, surface-free energy, chemical properties, and polymorphic transformation of minerals may result from comminution caused by changes in particle size and shape^25^. The shape of milled products varies according to the various ores’ breaking and grinding methods^26^. Unquestionably, the kind of grinding and its characteristics have an impact on the breaking approaches^27^. The size distribution and morphology of milled particles vary depending on the ore and mill parameters, including speed, loading ratio, ore type, and load size, feed size distribution, type of grinding circuit (wet/dry), and grinding time^28–33^.

One of the most precise techniques for determining the grinding energy and particle size distribution (PSD) is fractal geometry. A novel approach to studying the irregularity and complexity of nature is called fractal geometry^34^. Being a scale-independent constant, the fractal dimension offers clear benefits for characterizing data across several scales. Fractal characterization techniques for aggregate microstructure, surface morphology, and particle size distribution have been developed by researchers^35,36^. Using fractal geometry, Tasdemir has examined the distribution of product particle sizes after the comminution of several chromite ores using various tools. The findings demonstrated that the fractal dimension of each ore varies depending on the kind of comminution tools used, and that equipment type also significantly affects particle reduction and fractal dimension^37^. A study examined the impact of alteration of distinct ores and the sizes of feed particles. The findings demonstrated that the fractal dimension and the number of fine particles produced by crushing both increase with an increase in ores alteration and feed particle size^38^. The fractal dimension of the product particle grows and reduces with lowering setting size and rising feeding rate, respectively, according to research done on the impact of the jaw crusher’s feeding rate and setting size^39^. The studies used fractal geometry and the Rosin–Rammler model to characterize the size distribution of the crushed particles. Fractal geometry and Rosin–Rammler approaches were compared using the statistical index of root mean square error. The data collected indicate that the Rosin–Rammler approach is not as effective as the fractal geometry method^40–43^. Particle size distribution with fractal geometry was studied for permitted and unpermitted feeding in crushers. According to the results, the permitted and unpermitted capacities have a direct and inverse connection with the fractal dimension^44^. The impact of ore type and dry ball mill feed particle size on product particle size distribution was examined using fractal geometry. Based on the data gathered, it was discovered that the fractal dimension of the particle size distribution that results from grinding increases as the particle size of the coarser feed increases. Additionally, the type of ore significantly influences the fractal dimension and particle size distribution^45,46^. Fractal geometry was used to study the impact of wet and dry mills on the size and shape distribution of crushed particles. However, it was also looked into how these variables affected the magnetic separator’s performance. The outcomes demonstrated that the dry mill produced particles with bigger fractal dimensions and higher-grade magnetic separator products^47–49^. Another study looked at how wet grinding affected the morphology of particles with fractal geometry and how modifications to particle morphology affected the Denver mechanical flotation cell’s performance. The findings demonstrated a strong correlation between the flotation recovery and grade metrics and the fractal dimension of the particles^50^.

Fractal dimension is inversely related to particle size. Also, power draw is inversely related to particle size. Therefore, if the fractal dimension due to the particle size distribution increases while the power consumption decreases or remains constant, energy optimization has been successful. Chaotic grinding circuit prediction and optimization may be accomplished with the help of fractal dimension, a novel technique for characterizing and forecasting complicated and chaotic systems. However, the fractal dimension is a technique that can analyze and compute the specifics and the impact of small adjustments to each parameter on the system as a whole. In light of the previously described benefits, fractal dimension has been employed in this study to optimize the grinding procedure.

This study aims to investigate the particle size distribution of the dry magnetic separators tailing, which are fed into the regrinding mill of Gol Gohar iron ore, Sirjan, acquired by laboratory ball milling. The present study examines the correlation among fractal geometry, milling parameters, power draw, and energy consumption. Now that a logical relationship between these factors has been established, fractal geometry may describe the power draw of grinding circuits.

## Materials and methods

### Identification of samples

The sample taken from the regrinding circuit feed of Gol Gohar Sirjan, has been identified using particle size distribution, mineralogical analysis, and chemical analysis techniques.

#### Particle size distribution

**Fig. 1:**
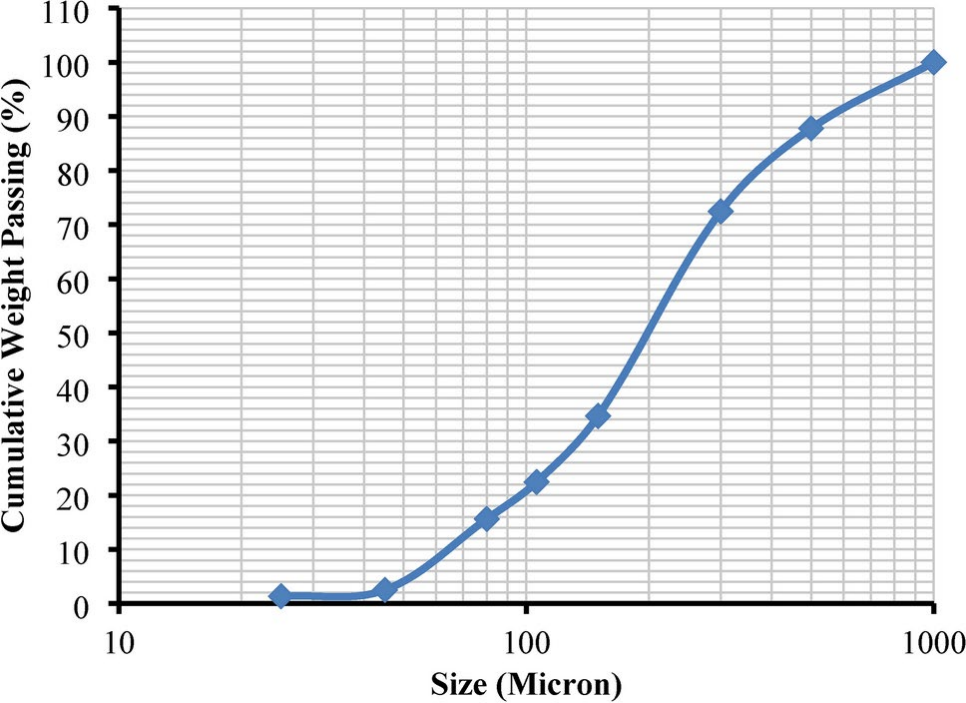
Particle size distribution analysis of the sample.

Figure 1 shows the particle size distribution of the sample, that its D80 is around 380 microns.

#### Mineralogical analysis

The X-ray diffraction (XRD) has been used to investigate the sample’s mineralogical properties. The analysis’s findings are displayed in Table 1. The findings indicate that magnetite, pyrite, and lizardite are the sample’s primary minerals.

**Table 1:** Mineralogical results by X-ray diffraction (XRD).

Mineral	Chemical formula
Magnetite	Fe_3_O_4_
Pyrite	FeS_2_
Lizardite	Mg_3_Si_2_O_5_(OH)_4_

#### Chemical analysis

The X-ray fluorescence (XRF) method was used to assess the chemical composition of the sample; the findings are shown in Table 2. According to the data, Fe_2_O_3_, SiO_2_, and MgO have the highest concentrations among the sample’s ingredients.

**Table 2:** Identification of chemical compounds of the sample by X-ray fluorescence (XRF).

Element	MgO	Al_2_O_3_	SiO_2_	S	CaO	Fe_2_O_3_	Other	L.O.I
Percentage	12.4	2.21	23.1	2.63	3.70	48.7	2.31	4.95

## Research method

### Milling

**Fig. 2:**
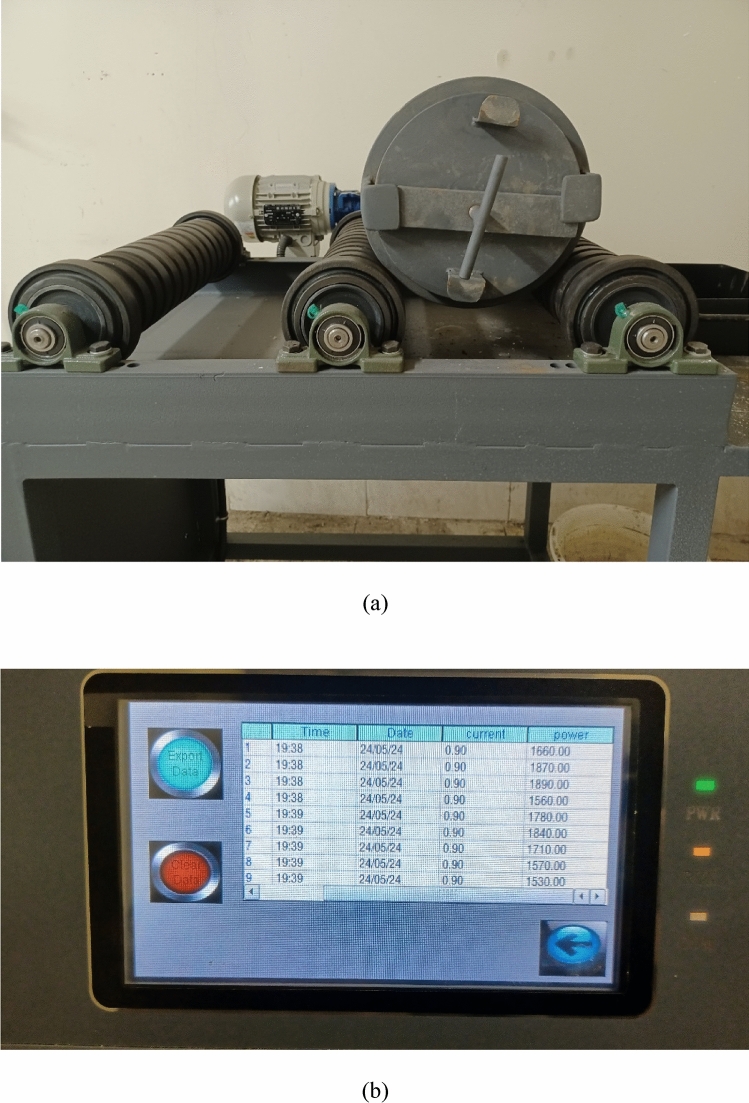
A picture of a used ball mill jar (**a**) and an online tool for recording power draw over time (**b**).

A ball mill jar (Fig. 2a) is employed in this study. The apparatus is outfitted with a system (Fig. 2b) that has captured all of the data online. The power drawing system records data about the mill’s power draw every 10 s. Each test was completed in 10 min, and sixty power draw data points were recorded for each test. The ball mill’s and the feed’s specs are displayed in Table 3. In contrast, the size distribution of the milled particles was examined using 300, 150, 106, 90, 83, 75, 63, 53, 45, 37, and 25 micron sieves. The sieves used were based on ASTM standards.

**Table 3:** Specifications of ball mill and feed.

Mill	Inner diameter, D (mm)	200
	Length, L (mm)	200
	Volume, V (cm3)	6283
	Critical speed, Nc (rpm)	94
	Operational speed (70% of critical speed) (rpm)	66
	Void between balls (cm3)	276, 352,427, 503, 578
	U (particle filling) (%)	40, 60, 80, 100, 120
Ball	Material	Manganese Steel
	diameter (mm)	12.7, 19.05, 25.4, 31.75, 38.1
	Specific gravity (gr/cm^3^)	7.5
Feed material	Specific gravity (gr/cm^3^)	4.12
	Feed size (mm)	0–1

The parameters of ball size, particle filling, and fractional ball filling have all been examined in this study. To compute the fractional ball filling, use formula 1^37^.

1 $${\text{J}} = \left[ {\frac{{\left( {mass\;of\;balls/ball\;density} \right)}}{{\left( {mill\;volume} \right)}}} \right]*\left[ {\frac{1}{0.6}} \right]$$

This study has examined the fractional ball filling of 11, 14, 17, 20, and 23 percent.

To compute the particle filling, formula 2 was used to determine the volume of the void between the balls. The amount of water and sample needed for each test was then estimated by calculating the solid weight percentage and the specific gravity of the feed. In every test, the solid’s weight percentage was 45%.

2 $$Void\;between\;balls = \left[ {J*\left( {mill\;volume} \right)} \right] - \left[ {volume\;of\;balls} \right]$$

Investigations have been conducted on particle filling at percentages of 40, 60, 80, 100, and 120. Additionally, balls of 12.7, 19.05, 25.4, 31.75, and 38.1 mm in diameter were employed.

### Fractal approach

One technique that can be applied to the linearization and description of particle size distributions is fractal geometry. A link between particle size and weight is established using formula (3) in order to examine the particle size distribution using fractal geometry^51–54^:

3 $$\frac{M(X < x)}{{M_{T} }} = \left( {\frac{x}{{X_{L} }}} \right)^{{3 - D_{f} }}$$

where M_T_ is the weight of the entire sample, x is the size of each dimensional fraction, X_L_ is the size of the greatest dimensional fraction, and D_f_ is the fractal dimension. M(X < x) represents the cumulative weight of particles passing through each dimensional fraction. For a scale-invariant (fractal) PSD, the logarithmic transformation of relationship (3) yields a linear connection between M(X < x)/M_T_ and x/X_L_:

4 $$ln\frac{{M\left( {X < x} \right)}}{{M_{T} }} = \left( {3 - D_{f} } \right){\text{ln}}\left( {\frac{x}{{X_{L} }}} \right)$$

D_f_ is calculated using the equation and the slope coefficient (m), for the linear regression line, was obtained from the linear best fit of the relationship (4).

5 $$D_{f} = 3 - m$$

This method limits the fragmentation’s fractal dimension to (0 < D_f_ < 3). The fractal dimension can be found using this method, which produces a comminution measure. The experimental situations of comminution are usually intermediate (D_f_≈2.5)^55,56^. Comminution studies usually yield values within the 2 < D_f_ < 3 range, with very infrequent values falling outside of this range. Consequently, the energy dissipation will occur in a fractal domain between a volume (D_f_ = 3) and a surface (D_f_ = 2). Thus, it is clear that the self-similarity of the pieces at all sizes indicates that energy is lost in a fractal set with a fractal dimension (D_f_) of (2 and 3)^57^.

A specific cumulative mass-size distribution is shown by each value of D_f_^58^. The value of D_f_fluctuates as a result of stress variations, material characteristics, and fracture mechanics. In grinding, the fractal dimension of the particle size distribution grows as the number of fine particles, the degree to which particles are broken up, and the spread of particle sizes all grow^59^. D_f_ is sensitive to changes in the process parameters and rises in value as the degree of particle comminution increases. While well-graded mixes with a range of sizes have higher values of D_f_, mixtures with uniform sizes that are dominated by particles of the original size have lower values^58^.

The broad PSD suggested by the Gates–Gaudin–Schuhman (GGS) distribution function is comparable in form to the relationship (3): M = 100[(x/k)^a^], where k is the size parameter, a is the uniformity index, M is the cumulative percentage finer than x, and x is the screen size. This suggests that the GGS line’s slope “a” equals 3_D_f_and that the GGS equation is fractal^59,60^. According to theory, lower values of “a” would indicate fewer particles in the medium range, more coarse particles, and more fines^59^.

### Experimental design

This study employed the central composite method, a common response surface technique, in which the fineware was given three parameters across three surfaces. Ball size, particle filling, and fractional ball filling were taken into account when planning the tests, with fractal dimension and power draw variables serving as the response. Version v10.0.7.0 of the Design of Experiment fineware was utilized in this investigation. The central composite method was used to design twenty tests with six central points.

#### Analysis of experimental models

The power draw and fractal dimension results were fed into the fineware as responses. After that, an appropriate model for the response was discovered using the analysis of variance (ANOVA) method. The relevance of a model is justified by the values F and P. The model’s validity was assessed at a 95% confidence level using high values of F and low values of P. P values less than 0.05 signify the parameter influences the response^61^. The model applied by Tables 4 and 5 has been verified. The fractal dimension and power draw model, as well as the interactions between different parameters, are depicted in Eqs. 6 and 7, which are represented in terms of coded factors. The fractional ball filling parameter, A, the particle filling, B, and the ball size parameter, C, are represented in these equations. The outcomes of the power draw and fractal dimension are largely influenced by the parameters of fractional ball filling and particle filling, respectively, according to the formulae. The precision of the model is one of the key factors in the experimental design that is fine-tuned to guarantee the model. The model’s adequate precision must be higher than 4 to be guaranteed. As can be seen from Table 4’s results, both of the models are significant because their adequate precision values are higher than 4.

6 $$\begin{aligned} P.D & = + 38.97 + 0.68A - 0.048B - 0.2C - 0.2AB - 0.57AC + 0.49B \\ & \quad + 0.45A^{2} + 0.02B^{2} + 0.3C^{2} - 0.068A^{2} B - 1.05AB^{2} \\ \end{aligned}$$

7 $$\begin{aligned} D_{f} & = + 1.53 + 0.0015A - 0.033B - 0.018C - 0.004AB - 0.0015AC + 0.013BC \\ & \quad - 0.0007A^{2} + 0.005B^{2} - 0.008C^{2} - 0.013B^{2} C - 0.016A^{2} B^{2} \\ \end{aligned}$$

**Table 4:** The model’s accuracy coefficient for each of the examined samples.

Response	Accuracy coefficient		
Power draw	Adeq precision	Mean	Std. Dev
	14.833	39.59	0.32
	Adj R-squared	C.V	R-squared
	0.8891	0.81	0.9533
Fractal dimension	Adeq precision	Mean	Std. Dev
	12.194	1.52	0.017
	Adj R-squared	C.V	R-squared
	0.8474	1.13	0.9357

**Table 5:** ANOVA for the investigated sample’s responses model.

Response	ANOVA parameters			
Power draw	Source	Sum of squares	df	Lack of Fit
	Model	16.95	11	0.0638 (not significant)
	Mean square	P-value Prob > F	F-value	
	1.54	0.0004 (significant)	14.84	
Response Fractal dimension	Source	Sum of squares	df	Lack of Fit
	Model	0.034	11	0.7422 (not significant)
	Mean square	P-value Prob > F	F-value	
	0.0003	0.0013 (significant)	10.59	

#### Variance analysis

ANOVA is a technique that uses variance ratios to verify if a model is significant or not. The ANOVA for the investigated sample’s power draw and fractal dimension responses are shown in Table 5, demonstrating the model’s significance for these responses.

#### Model validation

The experimental design software uses a variety of techniques, such as real response values in comparison to anticipated values, to validate the model. A good correlation was found between the fitting lines in the fractal dimension plots and the actual power tensile responses and their predicted values, respectively. As a result, the response model is legitimate and normal. The diagrams were left out to keep the article from getting too long.

## Results and discussion

This study uses particle size distribution to examine the effects of three parameters: ball size (B.S), particle filling (P.F), and fractional ball filling (F.B.F), on power draw (PD) and the fractal dimension (D_F_) of ground particles. Each of these criteria is looked at separately in the following.

### The effect of fractional ball filling

**Fig. 3:**
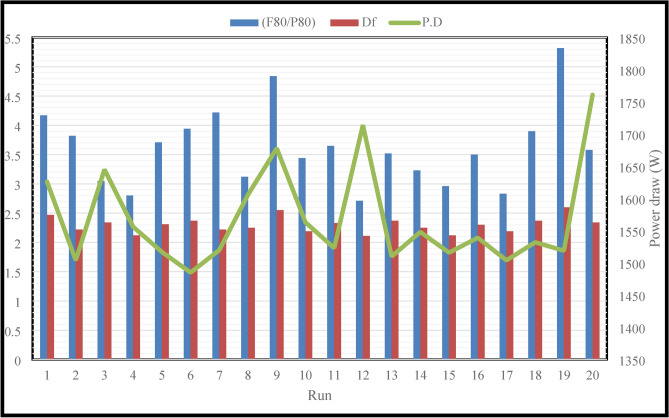
Variations in fractal dimensions (D_F_) and the ratio $\left( {\frac{F80}{{P80}}} \right)$ according to the power draw (PD).

This section looks into how the grinding mill’s fractional ball filling (F.B.F) affects the power draw (PD) and the fractal dimension (D_F_) of the ground particles, based on the PSD. Table 6 presents the overall findings. The outcomes of changes in the fractal dimension (D_F_) and the ratio $\left( {\frac{F80}{{P80}}} \right)$ depending on the power draw are displayed in Fig. 3.

**Table 6:** Overall findings of power draw and fractal dimension.

Run	F.B.F*	P.F*	B.S*	P.D* (W)	D_F_*	R^2^	$\frac{{F_{80} }}{{P_{80} }}$
1	14	60	19.05	1626	2.47	0.81	4.17
2	20	60	31.75	1504	2.22	0.86	3.82
3	17	80	12.7	1645	2.34	0.87	3.05
4	17	80	38.1	1556	2.12	0.94	2.80
5	17	80	25.4	1517	2.31	0.86	3.71
6	17	80	25.4	1485	2.37	0.86	3.94
7	17	80	25.4	1520	2.22	0.87	4.22
8	14	60	31.75	1606	2.28	0.90	3.12
9	20	60	19.05	1678	2.55	0.80	4.84
10	20	100	19.05	1563	2.19	0.87	3.44
11	17	80	25.4	1523	2.33	0.87	3.65
12	14	100	31.75	1714	2.11	0.95	2.71
13	17	80	25.4	1510	2.37	0.87	3.52
14	14	100	19.05	1548	2.25	0.88	3.23
15	20	100	31.75	1516	2.12	0.92	2.96
16	11	80	25.4	1539	2.3	0.88	3.50
17	17	120	25.4	1504	2.19	0.92	2.83
18	17	80	25.4	1532	2.37	0.84	3.90
19	17	40	25.4	1519	2.6	0.79	5.32
20	23	80	25.4	1761	2.34	0.87	3.58

*F.B.F = fractional ball filling, *P.F = particle filling, *B.S = ball size, *P.D = power draw, *D_F_ = fractal dimension.

**Fig. 4:**
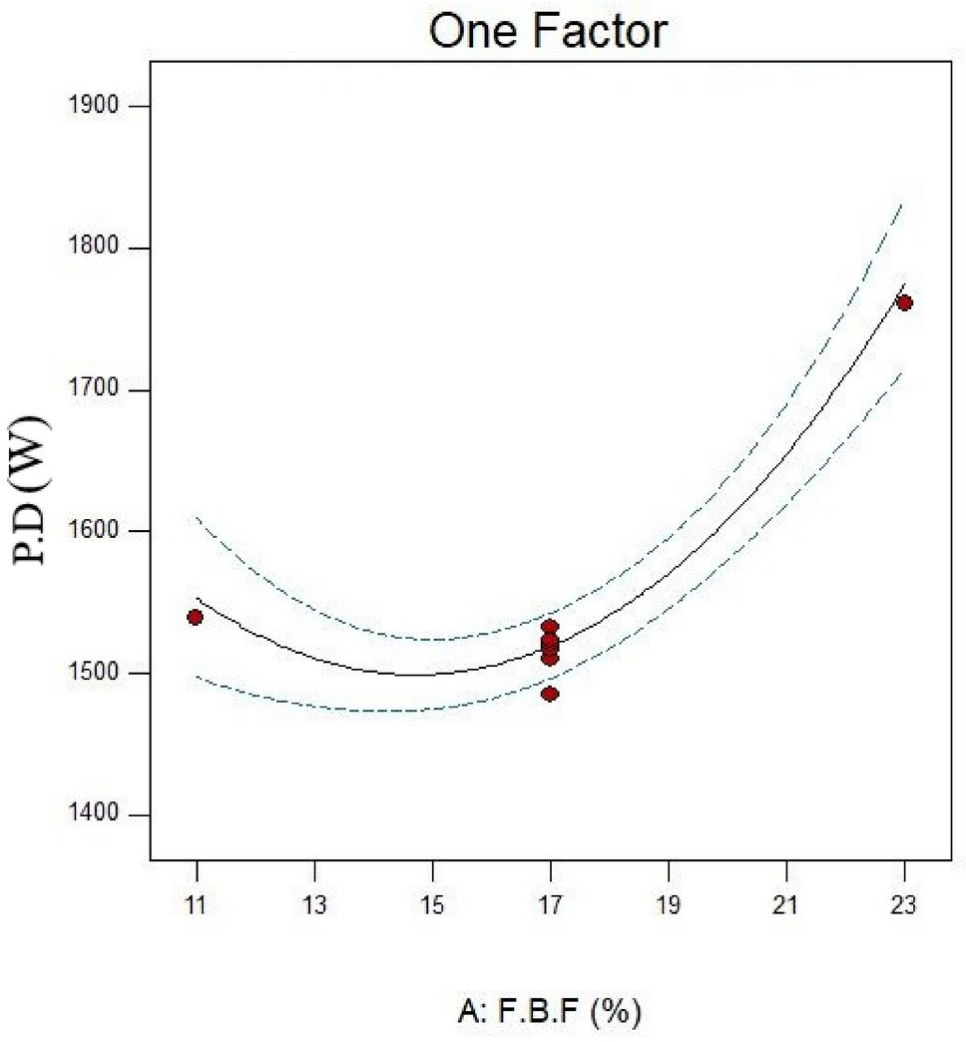
Variations in power draw (PD) according to variations in the fractional ball filling (F.B.F).

**Fig. 5:**
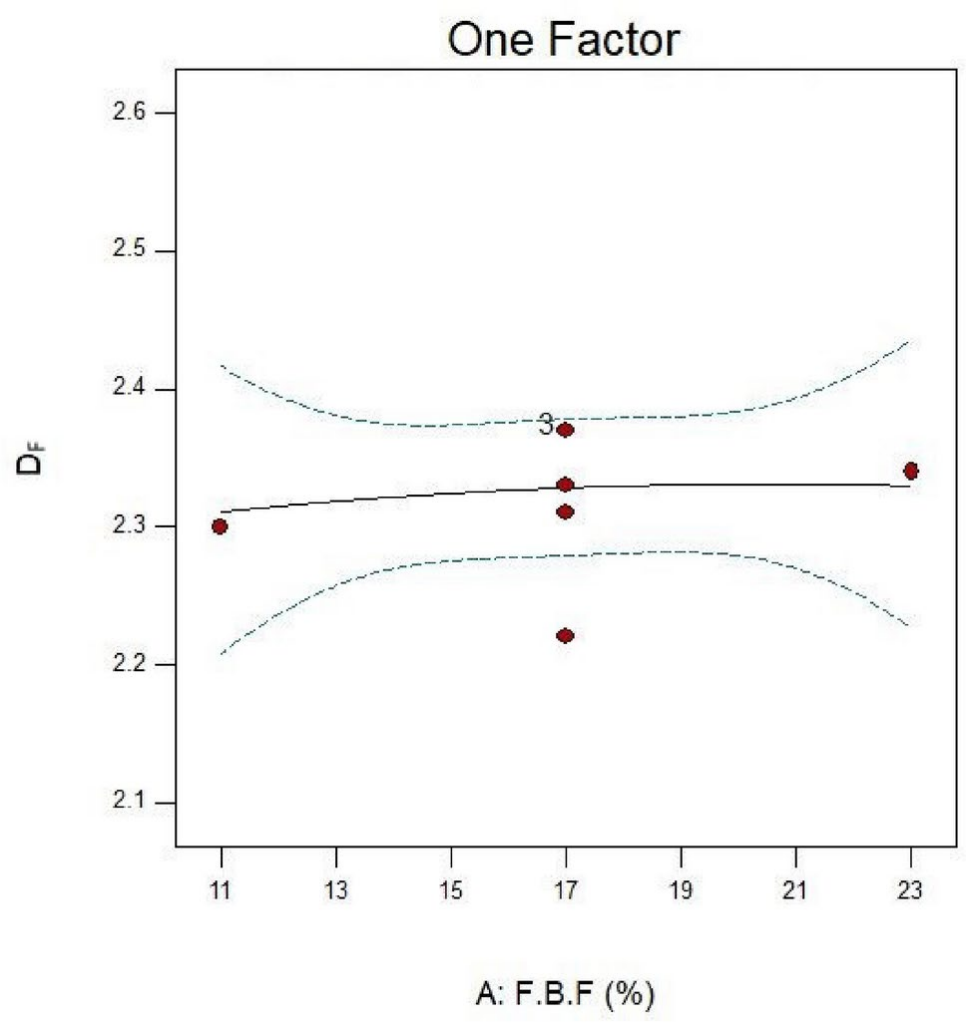
Fractal dimension (D_F_) variations for runs 13, 16, and 20 in connection to the fractional ball filling (F.B.F).

**Fig. 6:**
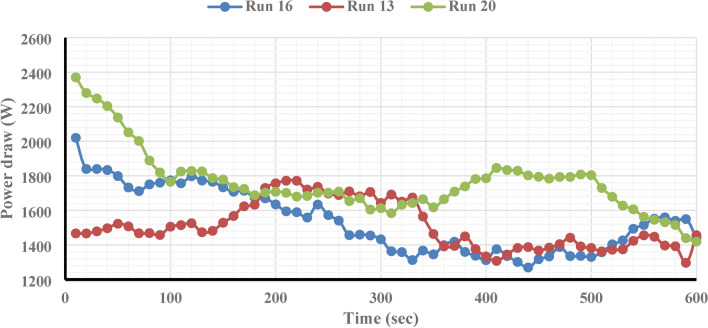
Evolution of power draw (PD) for runs 13, 16, and 20 over time.

This study employed F.B.F at 11, 14, 17, 20, and 23 percent. Runs 16, 13, and 20 in Table 6 were examined in more detail to investigate the effect of this parameter on power consumption, fractal dimension, and PSD while keeping other parameters constant. A closer look at Figs. 4 and 5 reveals that the PD and D_F_ slightly decrease and rise, respectively, when the F.B.F is increased from 11 to 17%. Table 6 and Fig. 3 findings make it evident that while fractional ball filling has increased, the ratio $\left( {\frac{F80}{{P80}}} \right)$ has only marginally increased as well. As a result, the D_F_ has likewise grown, indicating that the energy used is optimally used for particle further grinding. This could be because the balls are moving more uniformly and regularly as a result of the increased F.B.F, which has reduced energy usage. As the F.B.F lowers, the balls travel more erratically, which wastes more energy. Additionally, the power draw rises as a result of the balls colliding with one another and hitting the mill wall many times. The ball’s filling ratio went from 17 to 23%, which resulted in a notable increase in power draw as well as slight modifications to the ratio $\left( {\frac{F80}{{P80}}} \right)$and fractal dimension. This is because when F.B.F increases, the mill’s increasing weight and the balls’ transition from impact to abrasion movement mechanism greatly increase power draw. These variations clearly show that an increase in power draw will occur for extremely low or very high F.B.F percentages of the balls. This pattern of change aligns with earlier studies^62^. In contrast, the balls will move frequently, the grinding mechanism will switch from abrasion to impact, and the PD will be lower if the mill’s fractional ball loading is within the appropriate range, say 17%. The results of the time-dependent variations in PD for runs 13, 16, and 20 in Table 7 are displayed in Fig. 6. It should be noted that noises and mill movement may appear in the data within the first 100 s. This portion of the data has not been evaluated and will be thoroughly examined in the upcoming study. The tests with the highest power draw at the start were run 20 and 16, which saw a drop in PD after 80 s and an increase from 340 s forward. At the 500th second, however, run 20’s PD significantly decreases while run 16’s PD significantly rises. The grinding mechanism has switched from abrasion to impact and abrasion in the 20th run, when the mill reaches its maximum rotation speed and balances the movement of the ball load. Energy use is reduced as a result of this. Considering that this cycle took between 340 and 500 s to finish, it is expected to be repeated with longer grinding times. According to Fig. 6, comparing the 13th run to the other two, it was discovered that, in contrast, the run’s energy consumption began to drop after 340 s and continued to do so until the very end. These findings demonstrate that the fractional ball filling’s PD fluctuates at a rate of 17% less than that of 11% and 23%, which has resulted in a drop in PD. However, it should be highlighted that even though the power draw varied significantly, the D80 of the final products remained rather constant, yielding 107, 109, and 106 microns for runs 13, 16, and 20, respectively. Other studies have also shown that increasing tensile strength does not always result in finer particles^63^. Overall, the data suggests that it is possible to forecast the times during which PD will rise by learning more about the graph showing variations in energy use over time.

**Table 7:** Optimal conditions for grinding.

Parameter	Fractional ball filling (%)	Particle filling (%)	Ball size (mm)
Optimization	17.62	60	19.05
Power draw	1611		
Fractal dimension	2.57		
Desirability	0.928

**Fig. 7:**
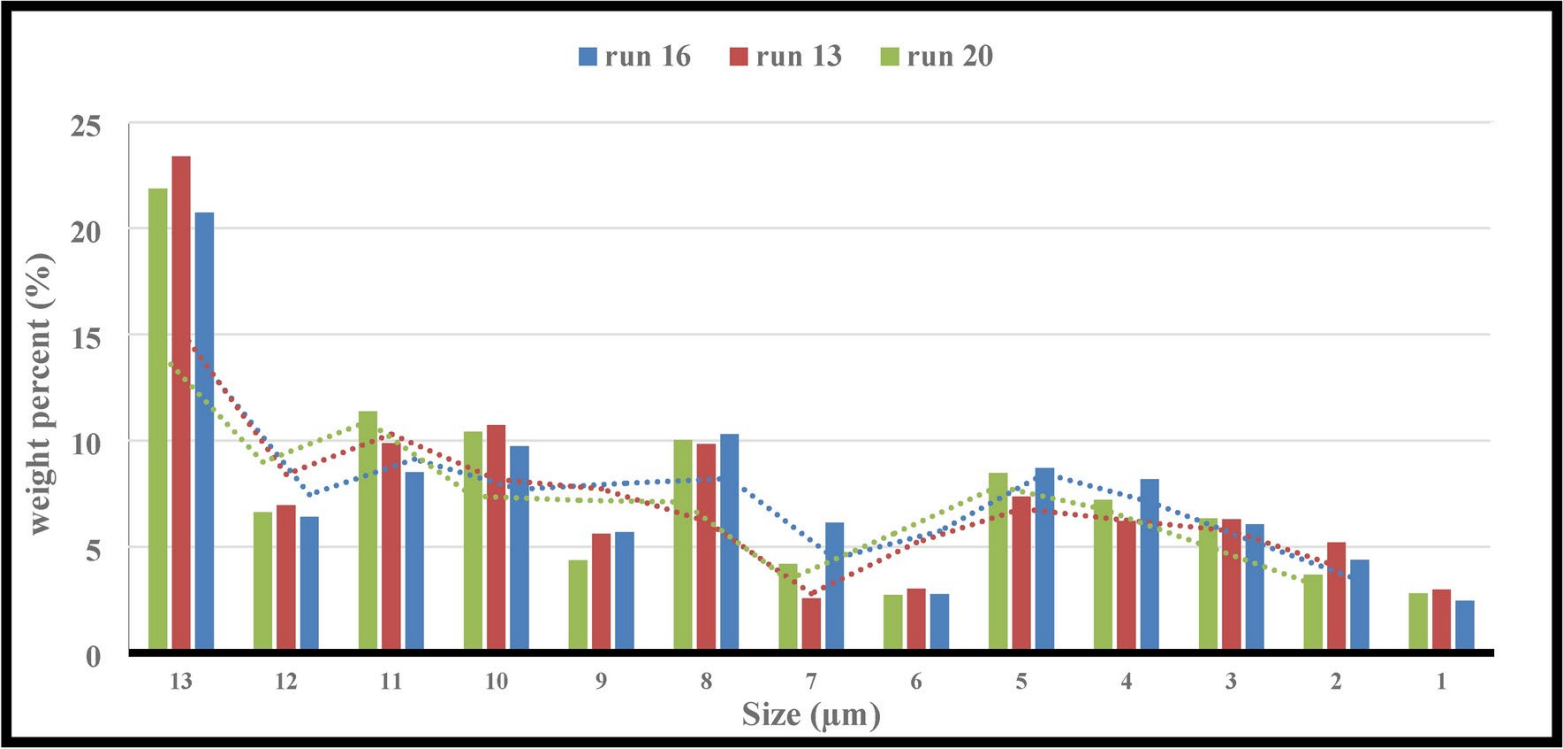
Weight percentages (runs of 13, 16, and 20) for various size fractions.

**Fig. 8:**
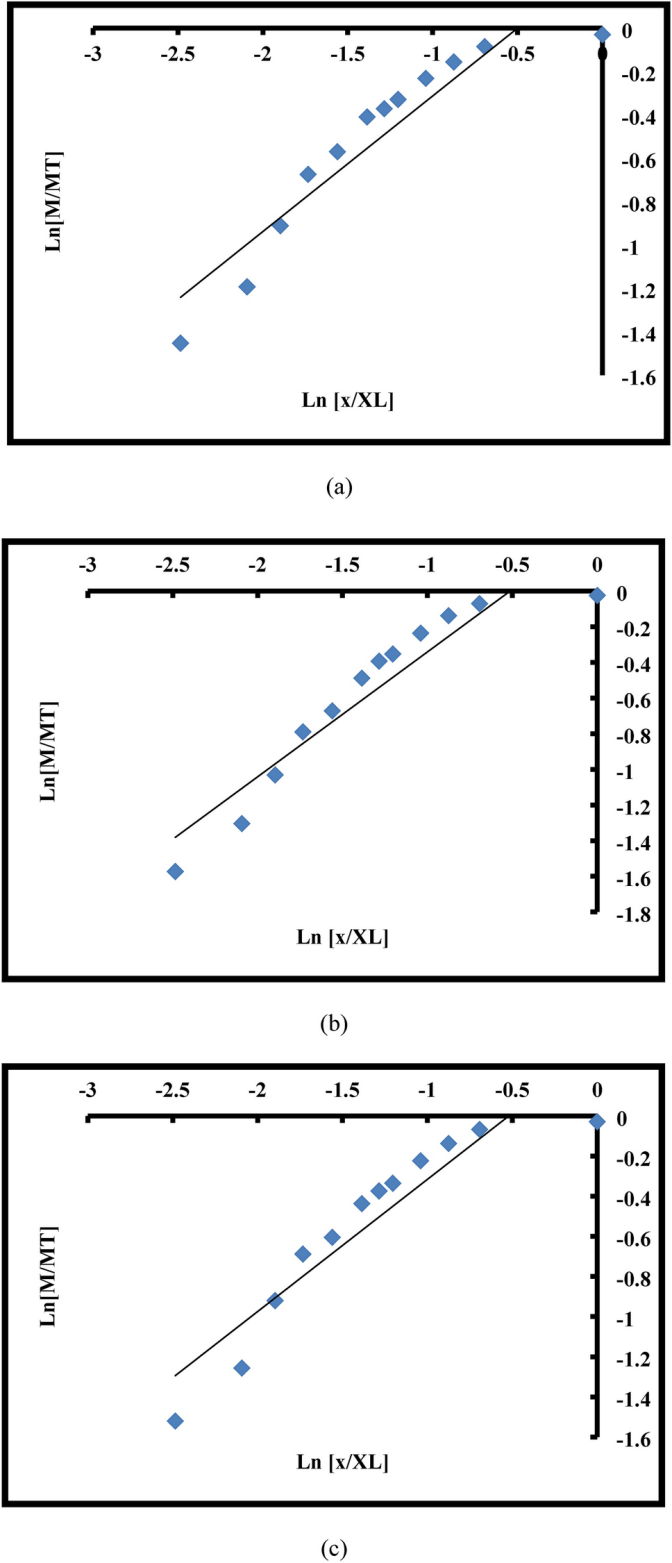
Fractal dimension (D_F_) graphs for runs 13 (**a**), 16 (**b**), and 20 (**c**).

However, given that the ratio $\left( {\frac{F80}{{P80}}} \right)$ and the D_F_ followed one another, it was discovered that grinding comparably altered the PSD (see Fig. 7). By monitoring these changes, the degree of liberation of the desired ore, and the self-similarity of the fractal geometry, it can forecast, manage, and optimize the granulation needed for mineral processing processes with the least amount of power draw. Consequently, by being aware of these variations, the effective parameters can be changed to shorten and lengthen the periods in which the PD rises and falls, respectively. Figures 8a, b, c displays the D_F_ graphs for runs 13, 16, and 20, respectively. To save the paper from getting too long, it was agreed not to include the fractal dimension graphs of the remaining runs.

### Effect of particle filling

**Fig. 9:**
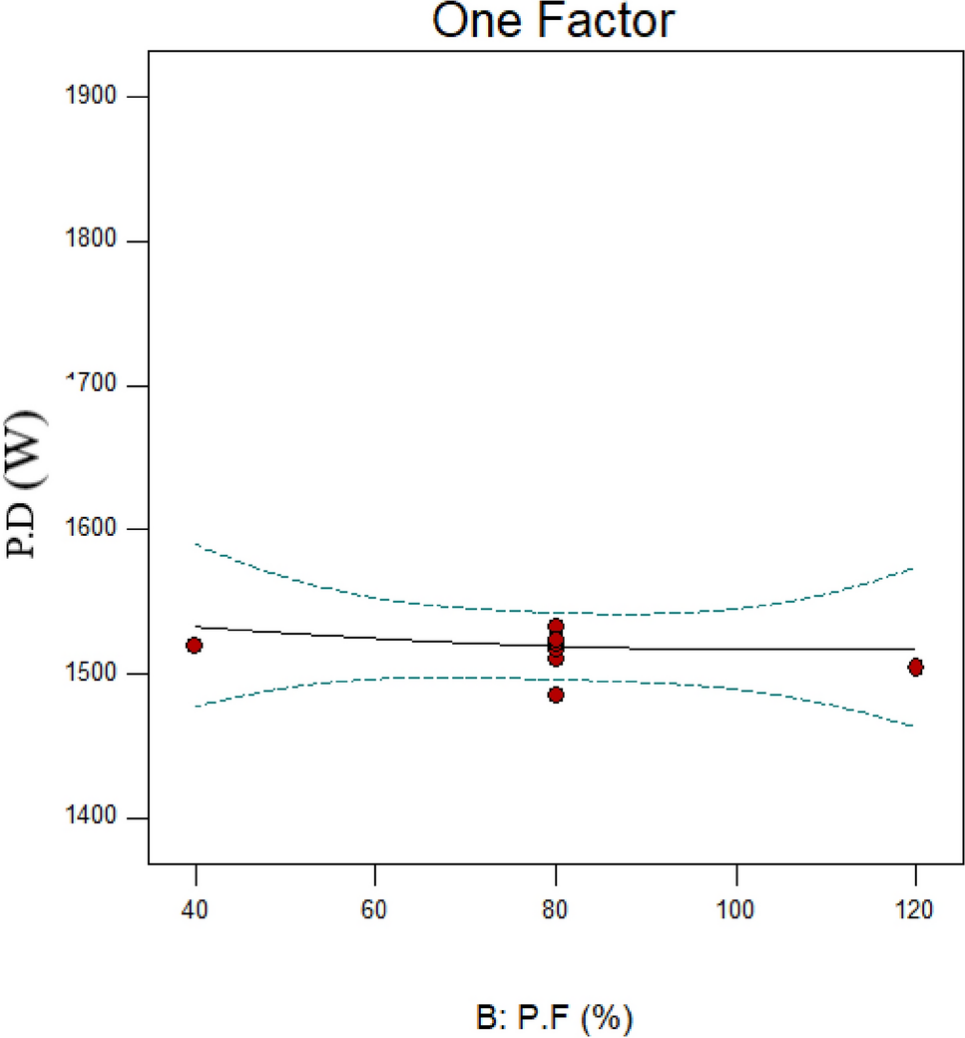
Variations in power draw (PD) according to variations in the particle filling (P.F).

The effects of particle filling (P.F) have been examined at percentages of 40, 60, 80, 100, and 120. Separate studies were conducted on the impact of this parameter on the D_F_ and PD. It is evident from Fig. 9—which displays the variations in PD concerning P.F—that the PD slightly reduces as the percentage of P.F increases. One possible explanation for this could be that as the P.F percentage increased, there was less of a ball-on-ball and wall collision^63^. This, in turn, reduced the electrical fluctuations in the mill’s electromotor, which further reduced the PD.

**Fig. 10:**
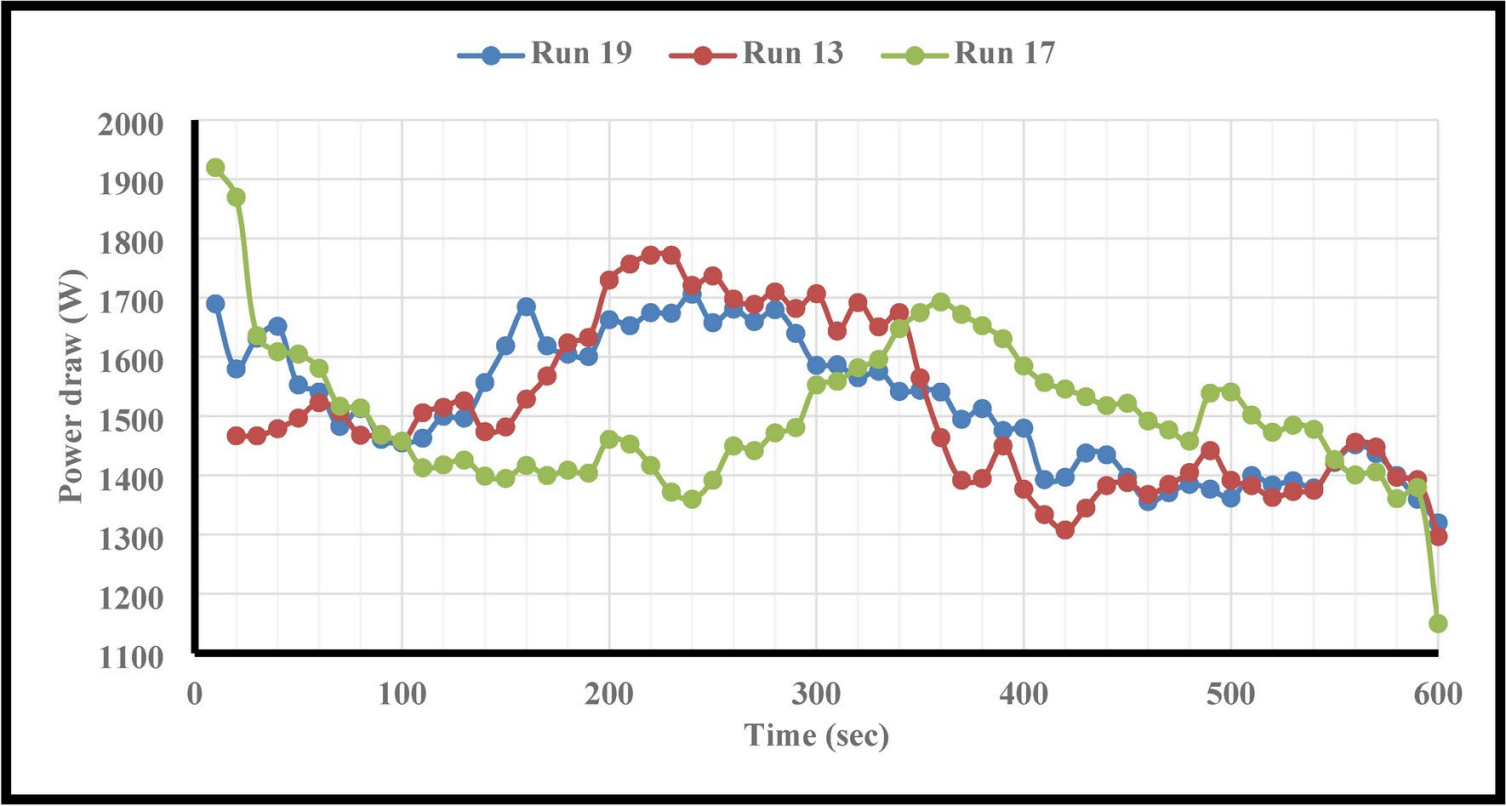
Evolution of power draw (PD) for runs 13, 17, and 19 over time.

The PD fluctuations over time for runs 13, 17, and 19 are displayed in Fig. 10. This graph demonstrates that, despite run 17 using more energy at first, this tendency has been declining throughout the run and has persisted through the first 240 s. After that, it continued to rise until the 360th second, at which point it started declining until the end. In run 19, the PD declined for the first 100 s, then increased until the 200th second, after which it was constant until the 300th second, then declining until the 400th second, and finally remaining constant until the conclusion. To avoid duplicating information, the modifications to the run 13 power draw trend have been thoroughly examined in the preceding section and are omitted here.

It is evident from the power draw data over time that modifications made inside the mill had an impact on energy usage. Thus, by regulating these changes and their affecting characteristics, it is feasible to decrease the periods during which the PD increases by knowing these changes, ultimately reducing the required PD. Rheology, particle size, ball size, pulp density, and other factors are some of the variables that alter during the softening process inside the mill^64^. It is anticipated that by adjusting these parameters, the required granulation will be produced faster and with reduced PD. It should be highlighted that although the PD has barely changed, the P.F has caused a significant change in the D80 of the product. Runs 13, 17, and 19 had D80 values of 107, 111, and 73 microns, in that order. However, it should be highlighted that the mill’s capacity in terms of tonnage has dropped to one-third as compared to run 17, even while running 13’s, D80 has fallen and its power draw has increased.

**Fig. 11:**
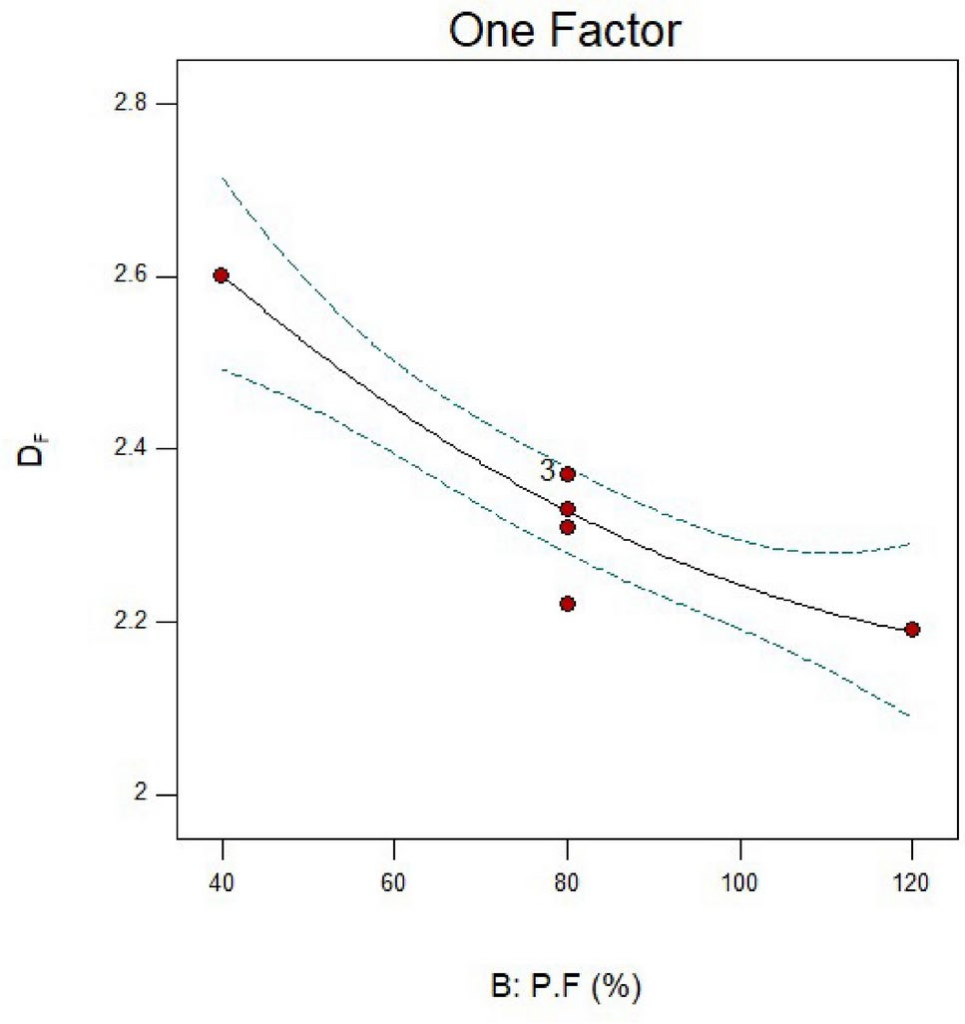
Fractal dimension (D_F_) variations for runs 13, 17, and 19 in connection to the particles filling (P.F).

The findings demonstrate that as P.F increases, the D_F_ significantly decreases (Fig. 11). As can be seen from Table 6 and Fig. 3 data, increasing P.F significantly lowers the ratio $\frac{F80}{{P80}}$ (from 5.32 to 2.83), which is directly correlated with changes in the D_F_. Other research shows that excessive particle filling reduces particle grinding and produces coarser particles^63–65^. It should be highlighted that the PD varies very little, indicating that the energy consumed is better utilized for crushing particles and is not wasted. This is because there has been less grinding of the particles due to the increased filling of the particles, which has reduced the weight percent of fine particles (25 microns) produced. Since the dispersion of the PSD and the rise in fine particles are directly correlated with the D_F_, the D_F_ has also decreased.

**Fig. 12:**
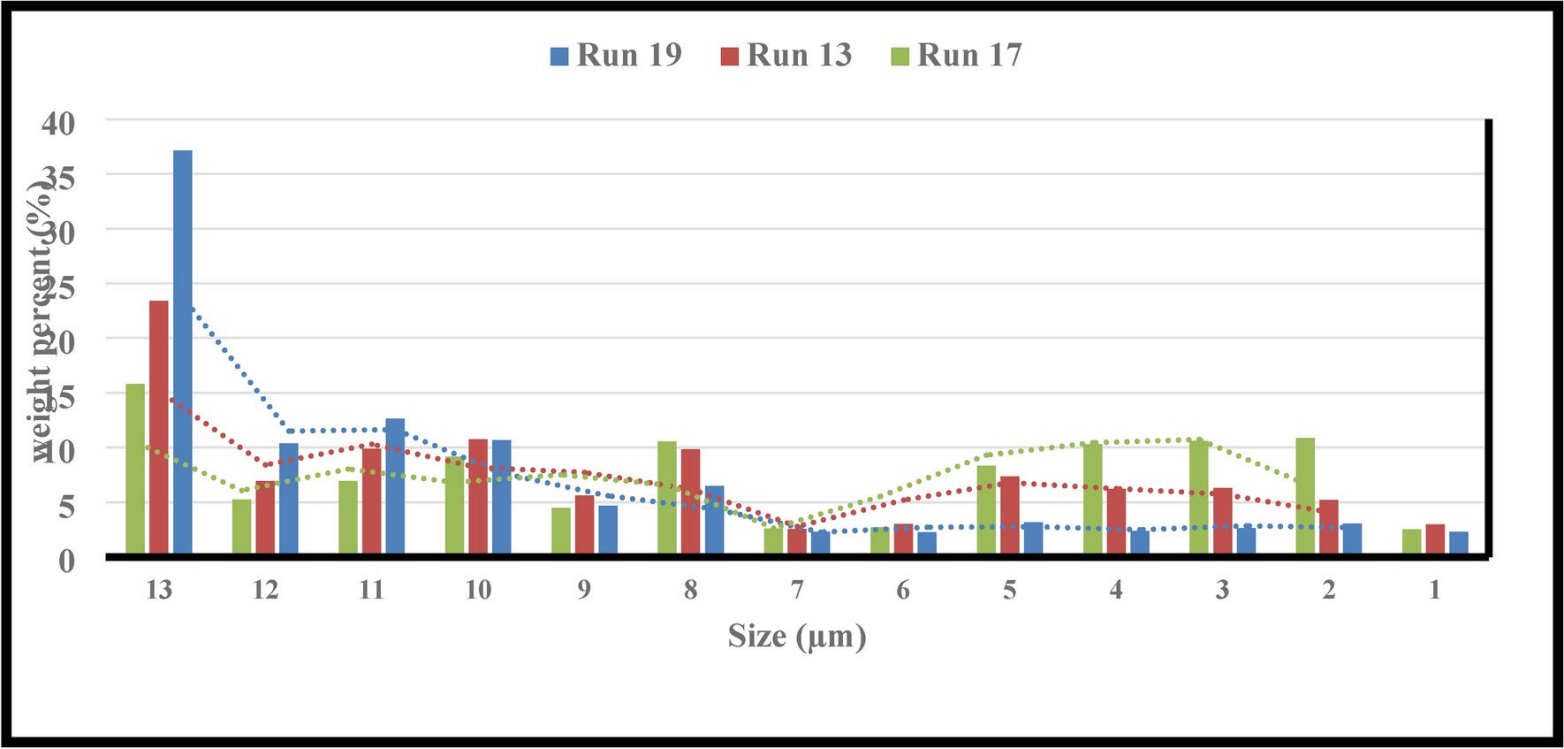
Weight percentages (runs of 13, 17, and 19) for various size fractions.

Figure 12 makes it evident that the number of particles smaller than 25 microns and the filling of particles have an inverse connection, with the amount of this fraction increasing significantly as the filling of particles decreases. It follows that a significant rise in the amount of D_F_ is anticipated. The possibility of more collisions between balls and particles has increased, which could be the cause of this, given that the filling of particles has been reduced while maintaining other parameters constant. Within the ball mill, coarse and fine particles can simultaneously make fine particles because all sizes of particles are crushed simultaneously. Thus, as the number of collisions rises, so does the crushing rate for all particle sizes, producing a greater quantity of fine particles.

### The effect of ball size

**Fig. 13:**
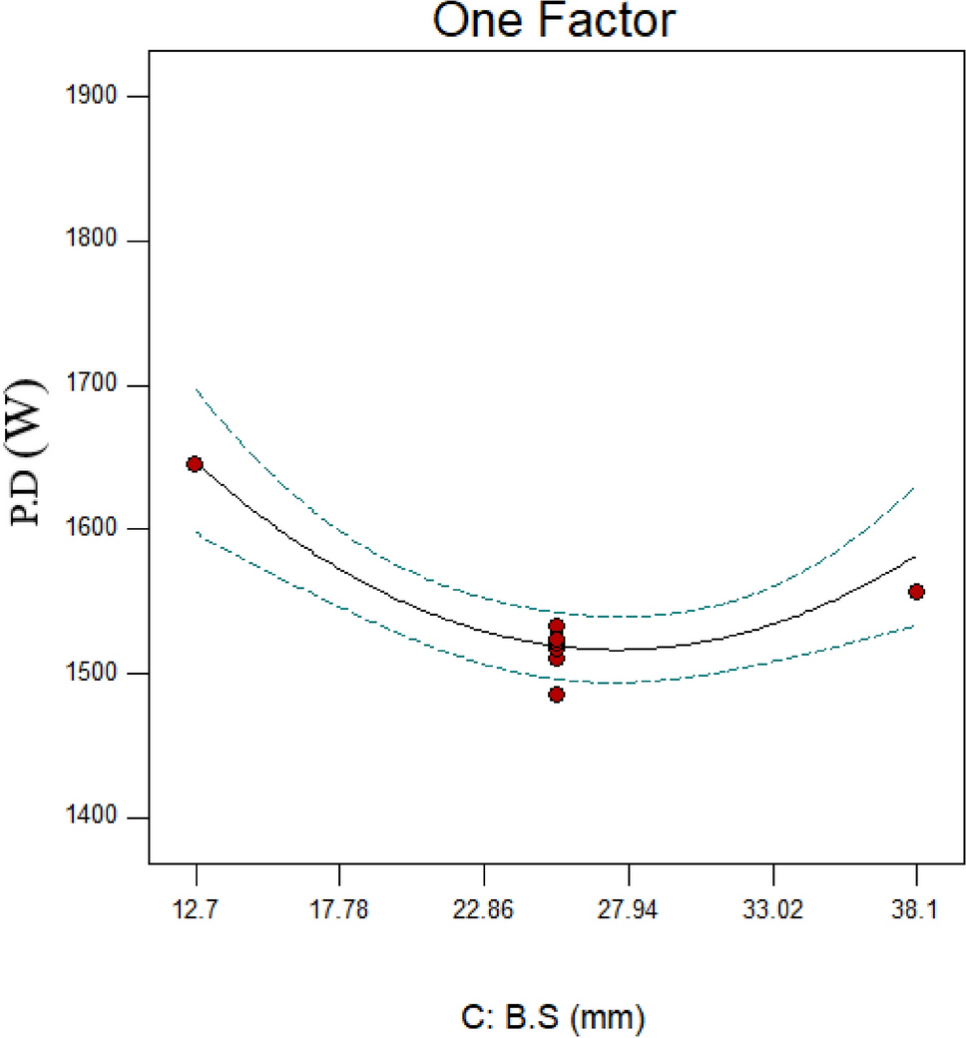
Variations in power draw (PD) according to variations in the ball size (B.S).

The size of the balls has been examined as one of the influencing aspects in this research since it affects the ball mill’s performance and is crucial to the particle grinding mechanism. Balls of 12.7, 19.05, 25.4, 31.75, and 38.1 mm are employed in this study as mono-size balls. The data shown in Fig. 13 demonstrate that the PD drops from 1645 to 1510 W (runs 3, 13, and 4 in Table 6) when the size of the balls grows from 12.7 to 25.4 mm. This could be because the number of balls decreased as a result of the balls’ increasing size owing to the fractional ball filling constant. This decreased the number of ball collisions with the mill wall and each other, which decreased the power draw. Conversely, the balls’ propensity to engage in toe movement as opposed to cascading increases as their size decreases. The grinding process shifts from impact to abrasion as the number of balls in the toe movement increases. Studies reveal that, compared to the impact mechanism, the abrasion process uses more energy. As a result, the use of balls with a smaller size (12.7 mm) may have increased PD due to a modification in the grinding process under these circumstances. The PD has increased from 1510 to 1556 W due to the balls’ size increase from 25.4 to 38.1 mm.

**Fig. 14:**
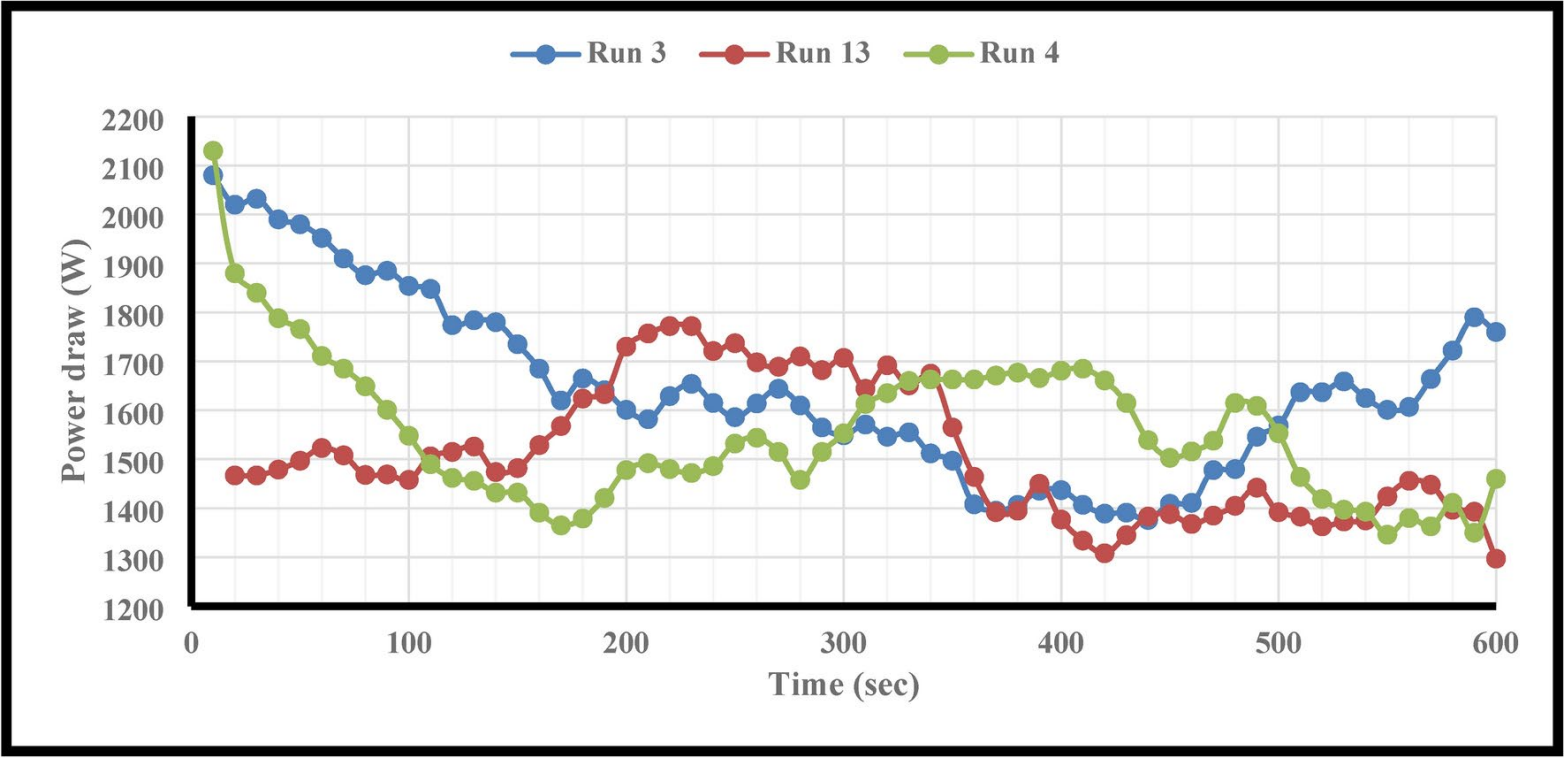
Evolution of power draw (PD) for runs 3, 4, and 13 over time.

One of the most significant causes of the rise in PD that corresponded with the size of the balls may have been the considerable irregularities in the ball movement at the start of the grinding process (refer to Fig. 14). However, coarse particles are often crushed using big balls^66,67^. If the particles are fine, the collisions between the balls have risen, resulting in a rise in PD over a period of time due to the change in particle size and the low contact surface of the balls with the particles. After 170 s, Fig. 14 makes this very visible. In the meantime, 440 s will pass if the balls have a size of 12.7 mm (see Fig. 14). Examining the case where the balls have a size of 25.4 mm reveals that this will occur after 140 s, but the increasing peak will last for 190 s. On the other hand, the balls whose sizes were 38.1 and 12.7 mm took 250 and 160 s to rise, respectively (see Fig. 14).

**Fig. 15:**
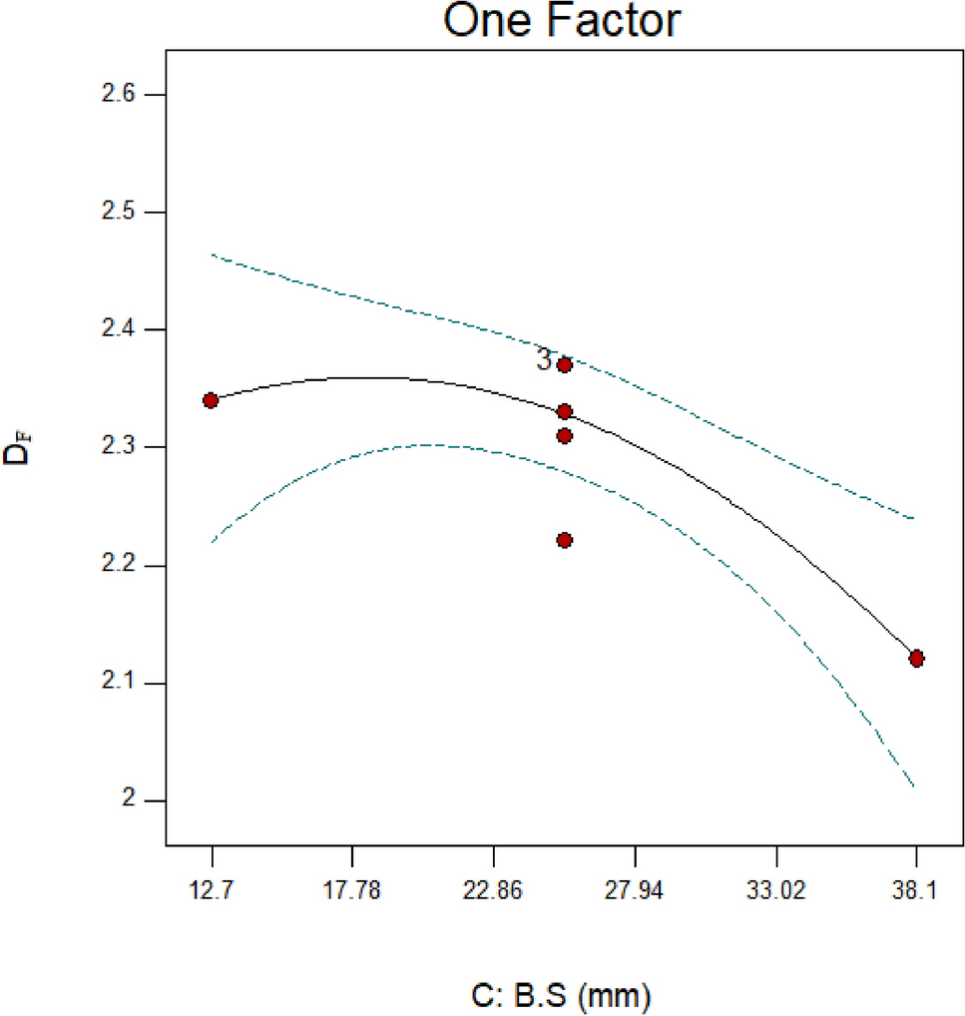
Fractal dimension (D_F_) variations for runs 3, 4, and 13 in connection to the ball size (B.S).

The outcomes of D_F_ for variations in B.S are displayed in Fig. 15. It is evident from Table 6 and Fig. 3 data that the ratio $\left( {\frac{F80}{{P80}}} \right)$ and the D_F_ are directly correlated. However, the rise in PD did not result in a further decrease in particle size and an increase in the ratio $\left( {\frac{F80}{{P80}}} \right)$. It is evident, though, that medium-sized balls (25.4) have the lowest energy loss and little balls (12.7) have the most energy loss. It is noteworthy that run 13, one of the six central points, is present. Table 6’s results make it evident that three of the central points have fractal dimensions of 2.37, meaning that they are higher than those of run 3. Reducing the size of the balls was not carried out in practice, despite the expectation that this would increase the amount of fine particles (finer than 25 microns).

**Fig. 16:**
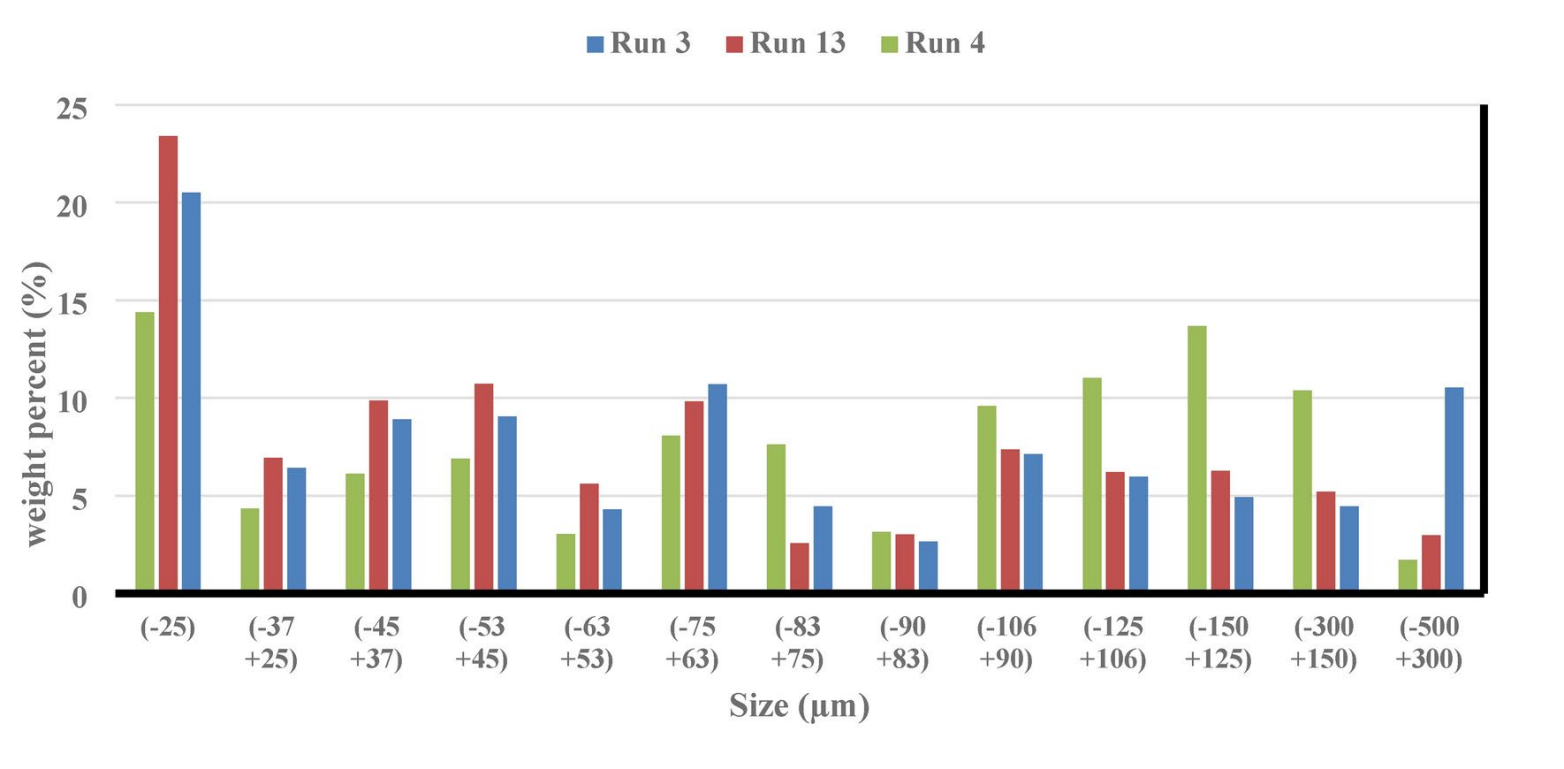
Weight percentages (runs of 3, 4, and 13) for various size fractions.

According to the findings, there were more fine particles in the scenario where the balls were 25.4 mm in diameter as opposed to 12.7 mm in diameter (see Fig. 16). The data obtained demonstrate that there are more particles coarser than 300 microns when the balls have a size of 12.7 mm. Therefore, it may be said that in this instance, the particle size distribution’s dispersion has risen. The DF has decreased significantly (from 2.37 to 2.12) by altering the ball diameters from 25.4 to 38.1 mm. By looking at Fig. 16, it is evident that 46.39% of the particles coarser than 90 microns and 14.39% of the particles finer than 25 microns were produced after the particles were grounded using balls the size of 38.1 mm. This is true even though the balls had a size of 25.4 mm, 28.04% of them were coarser than 90 microns, and 23.39% of them were finer than 25 microns. It has been previously studied that the size of the balls has a significant effect on the particle size distribution resulting from grinding^68^. Consequently, the decrease in the generation of small particles is the cause of the fractal dimension’s drop as the ball diameter increases. Based on these readings, it can be said that the dispersion of the particle size distribution is less influential on changes in the fractal dimension than the quantity of fine particles.

One may aspire for the optimization of grinding processes on an industrial scale as well, given the optimistic results of the fractal dimension technique. Notably, the scale remains constant in any situation, which is one of the fundamental tenets of fractal geometry. Stated differently, the outcomes derived from self-similar sets are scale-independent^51^. Thus, taking this concept into account, it can be said that this approach may also be successfully used on an industrial scale. To properly utilize fractal geometry, one must take into account its restrictions. Among the primary limitations of fractal geometry are the need for enormous amounts of data, the difficulty of understanding the information extracted from it, and the need for precise analytical instruments. Several factors need to be taken into account in order to implement this method on an industrial scale. These include the availability of precise digital equipment for analyzing the size distribution of the particles entering and exiting the mill, intelligent control of the mill’s operating parameters that can automatically apply changes to the operating parameters to optimize the grinding process based on the interpretation of the fractal dimension results, the presence of precise systems for calculating the mill’s power consumption, and the ability of the grinding operation operators to determine the target grain size based on the downstream needs.

### Interaction of parameters

**Fig. 17:**
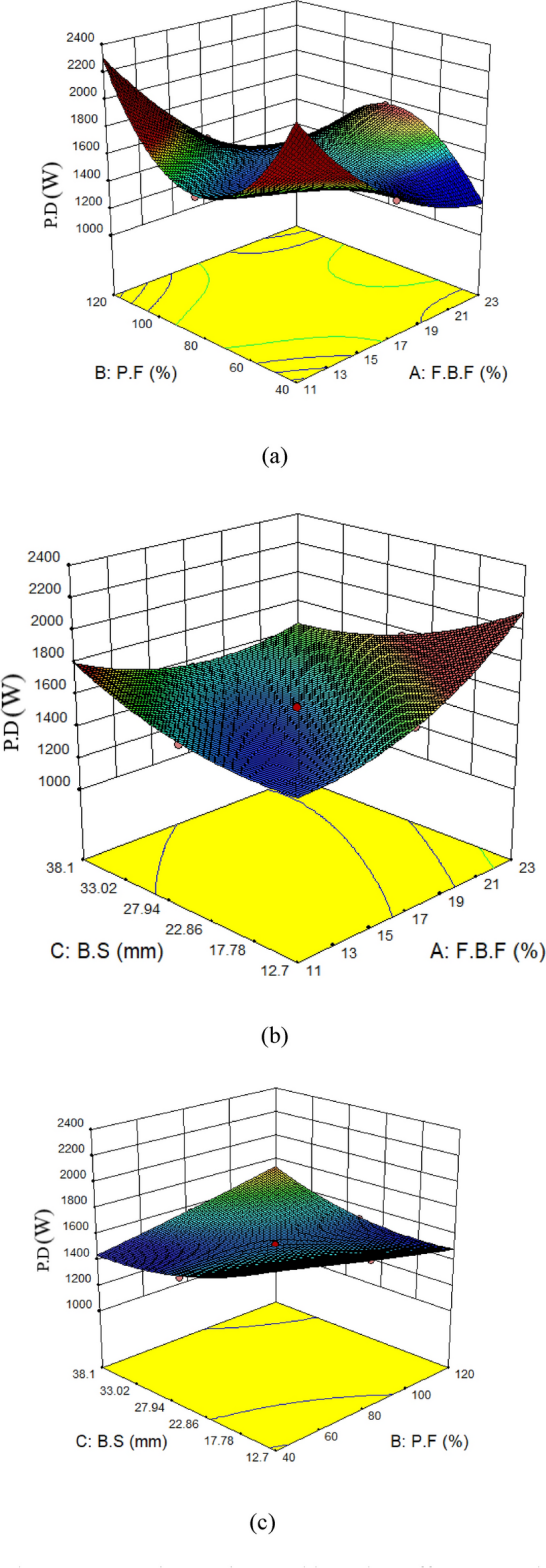
The parameters’ interactions and how they affect power draw (PD).

The effect of parameters interacting with one another has been examined in this section. The results of the parameter interaction on energy usage are displayed in Fig. 17 the findings demonstrate that an increase in PD occurs when both the F.B.F and P.F parameters are set at 11 and 40%, respectively. PD decreases initially and then increase with increasing P.F and constant F.B.F (11%); on the other hand, PD decreases with increasing F.B.F and constant P.F (40%). However, the result of PD will drop if both parameters are at their maximum state, which is 23% and 120%. However, the result shows that the lowest PD occurs when the F.B.F is 17% and the P.F is 80% (see Fig. 17a). The interaction between the B.S parameters and the F.B.F was then examined, as seen in Fig. 17b. The findings indicate that the amount of energy is reduced when the B.S is 12.7 mm and the F.B.F is 11%. The energy usage rises when the F.B.F is increased from 11 to 23% while maintaining a fixed B.S of 12.7 mm. Conversely, when the B.S is increased from 12.7 to 38.1 mm, the F.B.F remains constant at 11%, but the PD rises. The energy usage will, however, drop if both parameters are at their highest values. According to the interpretations, the state with the two parameters of the F.B.F and the B.S in the minimum state yields the lowest energy. The interaction between P.F and B.S is depicted in Fig. 17c. The PD will rise if the balls are 12.7 mm in size and the P.F is 40%. The energy usage will go down if the B.S (12.7 mm) is maintained and the P.F reaches 100%. On the other side, the PD will go down if the B.S increases to 38.1 mm and the filling percentage of particles remains at 40%. Energy use will rise if both parameters are at their highest values. It is evident from Fig. 17c that the PD is lowest when the P.F is 80% and the B.S is 27.94 mm.

**Fig. 18:**
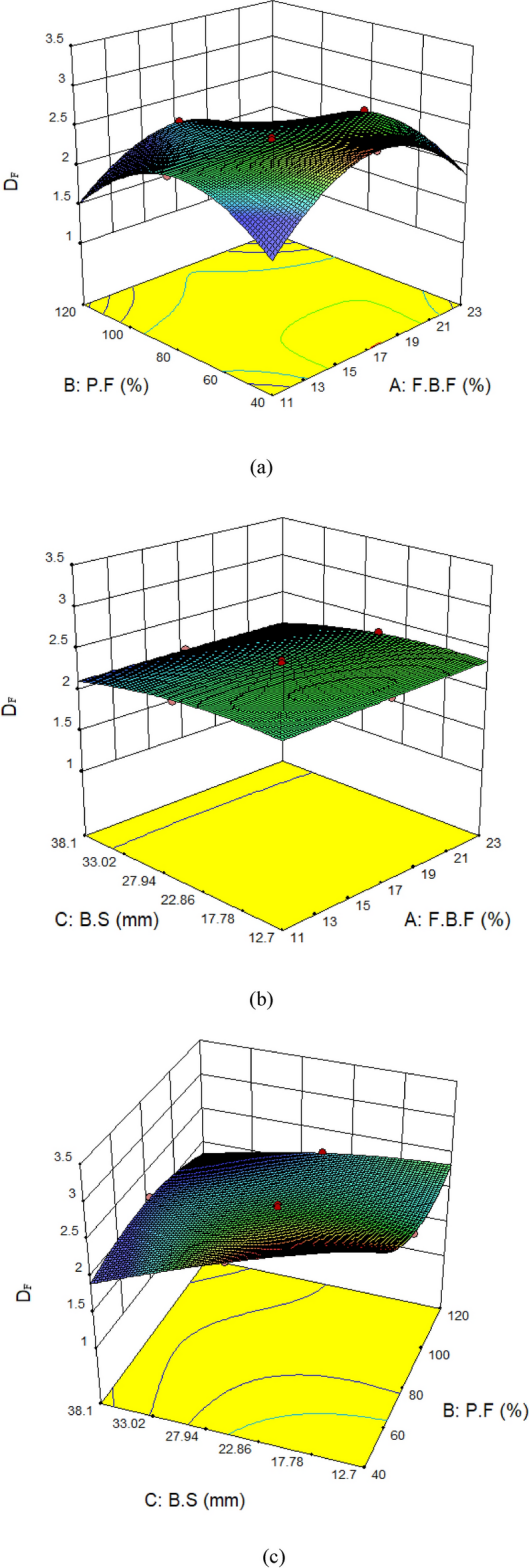
The parameters’ interactions and how they affect the fractal dimension (D_F_).

Figure 18 shows the parameters’ interactions with the D_F_. The relationship between the filling of the particles and the F.B.F in the D_F_ is presented in Fig. 18a. The findings indicate that the D_F_ falls with F.B.F and P.F of 11% and 40%, respectively. The D_F_ grows initially before decreasing if the F.B.F rises from 11 to 23% while the P.F remains constant. Conversely, the D_F_ grows and subsequently declines while the filling of balls remains constant and the filling of particles increases from 40 to 120%. Additionally, the D_F_ falls at the maximum values of both components. Since in grinding, the purpose is to generate more fine particles. It should be highlighted that there is a direct correlation between the D_F_ and the quantity of fine particles. Therefore, the objective has to be to increase the D_F_. Consequently, the D_F_ will be at its maximum when the F.B.F is 17% and the P.F is 40%. The interaction between the diameters of the balls and the F.B.F is examined in Fig. 18b. The findings demonstrate that the D_F_ does not alter when the F.B.F increases, while the B.S stay fixed at 12.7 mm. In contrast, the D_F_ has decreased if the F.B.F is 11% and the ball diameters are increased from 12.7 to 38.1 mm. If the balls have a size of 12.7 mm, the maximum D_F_ can be determined from the total filled fraction of the balls. Figure 18c illustrates the interaction between the ball diameters and the filling of particles. The findings indicate that the D_F_ will reach its maximum at a 40% P.F and 12.7 mm B.S. The D_F_ has shrunk while the B.S remain constant and the particle loading increases. On the other hand, when the balls got bigger and the P.F remained constant, the D_F_ shrank. The findings indicate that at maximal P.F and B.S, the lowest D_F_ is reached.

### Optimization

Table 7 displays the optimal results obtained through the experiment design software. Based on variance analysis, Eqs. (6) and (7) can be used to calculate the minimum and maximum values of power draw and fractal dimension. Ultimately, some optimal points are presented concerning the experimental model’s results. The largest fractal dimension was selected because the purpose of grinding is to produce more fine particles. The recommended ideal parameters are: 17.62% fractional ball filling, 60% particle filling, and a 19.05 mm ball size. The fractal dimension and the power draw prediction software in this case anticipated 2.57 and 1611, respectively. Table 8 presents the outcomes of a replication of the three tests. We’ll compare the actual outcomes with the forecasted ones. A model’s accuracy and correctness are indicated if the results are achieved within the required prediction range. There was a 95% confidence level in all results. Ultimately, the acquired outcomes validate both the model and the test outcomes.

**Table 8:** Results of actual and predicted optimal conditions.

Response	Number	Actual values	Difference estimated and experimental^a^	$\frac{a}{{\text{estimated }}}*100$ (%)	Predicted values at 95% confidence level
Power draw	1	1517	94	5.83	1574–1648
	2	1524	87	5.40	
	3	1545	66	4.10	
Fractal dimension	1	2.54	0.03	1.17	2.48–2.65
	2	2.56	0.01	0.39	
	3	2.53	0.04	1.56	

## Conclusion

Based on the modifications in the particle size distribution, the power draw and the fractal dimension of grinding were examined. The research’s overall findings are listed below.

- A large portion of the energy usage is determined by the ball load’s weight and mobility. The power draw has dropped when the fractional ball filling is increased from 11 to 17% since this decrease the quantity of unoccupied space inside the mill. Consequently, there has been a decrease in the irregularity of the ball load’s movement, which has decreased the power draw. Conversely, the energy consumed rises from 1510 to 1761 W when the fractional ball filling is increased to 23%. This is mostly because it takes more energy to rotate the mill due to its increased total weight. Another explanation for this is that when the area available for the ball load to travel is reduced, a lot of balls are positioned in the toe’s path. This causes the grinding mechanism to abrasion, which increases power draw.
- In the parameter of fractional ball filling, the maximal fractal dimension derived from the particle size distribution was seen when the fractional ball filling reached its middle stage.
- The power draw has decreased as a result of fewer ball impacts with the mill wall and with each other when the particle filling increases.
- The fractal dimension has expanded to 2.6 as a result of increasing the amount of fine particles produced by decreasing the particle filling to 40%. This represents the maximum fractal dimension found in this study. It should be noted that decreasing the particle filling will result in a decrease in the mill’s input tonnage. The mill has a one-third capacity, according to the results.
- The collected results indicate that the ratio $\left( {\frac{F80}{{P80}}} \right)$ and the fractal dimension are directly related.
- It’s evident from looking at power draw and ratio $\left( {\frac{F80}{{P80}}} \right)$ data that, for some parameters (like the size of the balls changed), a rise in power draw did not result in a corresponding increase in the ratio. This illustrates how adjusting a few parameters significantly lowers energy loss.
- A closer look at the size of the balls reveals that when they are small (12.7 mm), the contact surface, friction, and collision of the balls will rise due to the increased number of balls, which will increase power draw. Additionally, when more balls are involved in the toe’s movement, the grinding mechanism will shift from impact-abrasion to total abrasion, requiring more energy to operate. Additionally, when the balls get bigger—up to 38.1 mm in size—more energy is needed because of the disordered movement of the balls, which is brought on by their larger moving spaces. When examining the size of the balls, it is found that the best power draw occurs when the balls are of an intermediate size (25.4 mm).
- The fractal dimension went from 2.34 to 2.37 when the balls’ size was raised from 12.7 to 25.4 mm. This was not anticipated, as small (12.7 mm) balls are often well suited for creating fine particles. The quantity of fine particles generated with balls of 12.7 mm size was lower than with balls of 25.4 mm size, despite the increase in energy usage. The fractal dimension has been sharply reduced to 2.12 by increasing the size of the balls from 25.4 to 38.1 mm.
- The self-similarity principle of fractal geometry can be used to verify that power draw adjustments in a ball mill are repeatable. Therefore, even in cases where a linear link between power draw and fractal geometry cannot be identified, fractal geometry can be utilized to predict peaks of increase or decrease in power draw and to regulate the amount of energy consumed. The results collected indicate a similar and recurring pattern in energy use over time. Fractal geometry’s self-similarity principle can be used to forecast this behavior and develop an algorithm for it. Consequently, the time of the increasing phase of the power draw can be decreased or the effective parameters can be changed so that the power draw is always at its lowest value. This novel concept may be the basis for numerous studies in fractal geometry and energy usage.

## Data Availability

Upon reasonable request, the corresponding author will make the datasets used in this study available.
